# Lipopolysaccharide Induces Lung Fibroblast Proliferation through Toll-Like Receptor 4 Signaling and the Phosphoinositide3-Kinase-Akt Pathway

**DOI:** 10.1371/journal.pone.0035926

**Published:** 2012-04-26

**Authors:** Zhengyu He, Yuan Gao, Yuxiao Deng, Wen Li, Yongming Chen, Shunpeng Xing, Xianyuan Zhao, Jia Ding, Xiangrui Wang

**Affiliations:** Department of Anesthesiology, Renji Hospital, Shanghai Jiaotong University School of Medicine, Shanghai, China; Virgen Macarena University Hospital, School of Medicine, Spain

## Abstract

Pulmonary fibrosis is characterized by lung fibroblast proliferation and collagen secretion. In lipopolysaccharide (LPS)-induced acute lung injury (ALI), aberrant proliferation of lung fibroblasts is initiated in early disease stages, but the underlying mechanism remains unknown. In this study, we knocked down Toll-like receptor 4 (TLR4) expression in cultured mouse lung fibroblasts using TLR4-siRNA-lentivirus in order to investigate the effects of LPS challenge on lung fibroblast proliferation, phosphoinositide3-kinase (PI3K)-Akt pathway activation, and phosphatase and tensin homolog (PTEN) expression. Lung fibroblast proliferation, detected by BrdU assay, was unaffected by 1 mug/mL LPS challenge up to 24 hours, but at 72 hours, cell proliferation increased significantly. This proliferation was inhibited by siRNA-mediated TLR4 knockdown or treatment with the PI3K inhibitor, Ly294002. In addition, siRNA-mediated knockdown of TLR4 inhibited the LPS-induced up-regulation of TLR4, down-regulation of PTEN, and activation of the PI3K-Akt pathway (overexpression of phospho-Akt) at 72 hours, as detected by real-time PCR and Western blot analysis. Treatment with the PTEN inhibitor, bpV(phen), led to activation of the PI3K-Akt pathway. Neither the baseline expression nor LPS-induced down-regulation of PTEN in lung fibroblasts was influenced by PI3K activation state. PTEN inhibition was sufficient to exert the LPS effect on lung fibroblast proliferation, and PI3K-Akt pathway inhibition could reverse this process. Collectively, these results indicate that LPS can promote lung fibroblast proliferation *via* a TLR4 signaling mechanism that involves PTEN expression down-regulation and PI3K-Akt pathway activation. Moreover, PI3K-Akt pathway activation is a downstream effect of PTEN inhibition and plays a critical role in lung fibroblast proliferation. This mechanism could contribute to, and possibly accelerate, pulmonary fibrosis in the early stages of ALI/ARDS.

## Introduction

Pulmonary fibrosis is characterized by aberrant proliferation and activation of lung fibroblasts and collagen secretion that results in excessive extracellular matrix (ECM) deposition [1], [2]. The process of pulmonary fibrosis can be stimulated by pathogen infection, wherein the host cells recognize and respond to pathogen-associated molecular patterns (PAMPs). One of the most well characterized PAMPs is the lipopolysaccharide (LPS) component of the outer membrane of Gram-negative bacteria. LPS exerts its biological effects on the host by binding to Toll-like receptor 4 (TLR4), a pattern recognition receptor (PPR) that is widely distributed among lung parenchyma cells, including macrophages, epithelial cells, and fibroblasts. Our previous studies have shown that LPS plays an important role in the development of acute lung injury (ALI), acute respiratory distress syndrome (ARDS), and pulmonary fibrosis, through activation of TLR4 and its downstream intracellular signal transduction pathways [3], [4]. In addition, our study and those by others have also determined that focal aggregation of lung fibroblasts occurs prior to formation of fibrosis [5], implying that aberrant proliferation of fibroblasts takes place in the early stages of ALI/ARDS. However, the mechanisms underlying fibroblast proliferation remain unknown.

In another previous *in vitro* study from our lab, we determined that LPS was able to directly induce secretion of collagen in primary cultured mouse lung fibroblasts *via* TLR4-mediated activation of the phosphoinositide3-kinase-Akt (PI3K-Akt) pathway [6]. This finding raised the questions of whether LPS directly affects lung fibroblast proliferation, and which factors or signaling mechanisms are involved. Several recent studies have revealed that the PI3K-Akt pathway is closely related to cell cycle regulation [7], [8], [9]. The tumor suppressor phosphatase and tensin homolog (PTEN) acts to dephosphorylate phosphatidylinositol (3,4,5)-trisphosphate (PIP3), resulting in inhibition of the PI3K-Akt pathway. Low expression of PTEN and activation of the PI3K-Akt pathway have been found in lung fibroblasts from patients with idiopathic pulmonary fibrosis (IPF) [10], [11]; thus, we inferred that LPS may promote lung fibroblast proliferation by down-regulating PTEN expression and activating the PI3K-Akt pathway in the early stages of ALI/ARDS.

We used the knowledge and model system from our previous research to investigate this new hypothesis. Specifically, we knocked down TLR4 expression in cultured mouse lung fibroblasts using lentivirus-based RNA interference (RNAi), and then systematically evaluated the effect of LPS challenge on lung fibroblast proliferation, PTEN expression, and TLR4-mediated PI3K-Akt pathway activation.

## Materials and Methods

### Ethics statement

This study was approved by the Animal Care and Use Committee of the Shanghai Jiaotong University School of Medicine. All procedures were carried out in accordance with the guidelines for animal care published by the United States' National Institutes of Health (NIH) for animal care (Guide for the Care and Use of Laboratory Animals, Department of Health and Human Services, NIH Publication No. 86-23, revised 1985).

### Primary cultures of mouse lung fibroblasts

Lung fibroblasts were isolated from a C57/BL6 mouse, as described in our previous study [6]. Briefly, an eight-week-old mouse (Shanghai SLAC Laboratory Animal Co. Ltd., China) was sacrificed by decapitation. Lung tissues were promptly excised, washed with phosphate buffered saline (PBS), and cut to 1 mm^3^-sized tissue masses. Tissues were then distributed evenly along the bottom of culture plates and covered with Dulbecco's Modified Eagle's Medium (DMEM) containing 10% calf serum (Gibco, USA). The plates were cultured at 37°C in a humidified 5% CO2 incubator (Labotect, Germany), and DMEM was changed every three days. When the cultures reached 80% confluence, adherent cells were detached by exposure to 0.25% trypsin for five minutes, and then passaged at a 1∶4 dilution. Cells grew to a typical fusiform shape after four generations. Fibroblasts were characterized as previously described [12], and then used from passages five and seven.

### Construction and identification of RNAi lentivirus vector targeting the TLR4 gene

A TLR4-specific RNAi lentivirus vector (TLR4-siRNA-lentivirus) was constructed and verified by the Shanghai GeneChem Co. Ltd. (China), as described in our previous study [6].

### Experimental design and treatment

Purified mouse lung fibroblasts in DMEM containing 10% calf serum were seeded into 96-well plates and grown in a humidified atmosphere containing 5% CO_2_. When ∼60% confluence was reached, the medium was replaced with serum-free medium and the cultures were incubated for an additional 24 hours at 37°C in 5% CO_2_. Finally, the serum-free medium was replaced with DMEM containing 10% calf serum and the cells were divided among several groups for various experimental manipulations, as detailed below:

To inhibit expression of TLR4 mRNA in lung fibroblasts, TLR4-siRNA-lentivirus was added to cells at a concentration of 1×10^8^ TU/mL for 48 hours. Negative controls were established by adding the same doses of negative control-siRNA-lentivirus containing scrambled nonfunctional RNAi sequences. To inhibit activation of the PI3K-Akt pathway, the PI3K inhibitor Ly294002 (CST, USA) was added to cells at a concentration of 50 µmol/L and incubated for one hour. To inhibit activation of PTEN, cells were exposed to PTEN inhibitor (1 µM bpV(phen); Alexis, USA) for 0.5 hour. To investigate the effect of LPS challenge on lung fibroblast proliferation, purified LPS (derived from O55:B5 *E. coli*; Sigma, USA) was added to cells at a concentration of 1 µg/mL and incubated for 72 hours (or the time indicated in the figures). Experiments were performed three times in each group. Cells were collected for measurements at 72 hours (or the time indicated in the figures) after LPS stimulation. Cell proliferation was assessed by the BrdU assay. The mRNA and protein expression of TLR4, PTEN, and phosphorylated Akt were examined by real-time PCR or Western blot analysis, respectively.

### Cell proliferation assay

To evaluate lung fibroblast proliferation after different treatments, quantification of DNA synthesis, a direct indication of cell proliferation, was assessed by the BrdU cell proliferation assay, as previously described [11]. Briefly, prior to harvesting, cells were incubated with BrdU for four hours. Anti-BrdU antibody was applied as an immuno-stain, according to the manufacturer's instructions (CST). DNA synthesis was quantified by the magnitude of absorbance (optical density, OD) at 450 nm, which was proportional to the quantity of BrdU incorporated into cells.

### Real-time PCR

The mRNA expression of TLR4 and PTEN was analyzed by real-time PCR. Total RNA was isolated from cells with the RNeasy kit using the Trizol reagent (Invitrogen, USA) and was reverse-transcribed into cDNA with a reverse transcription kit using M-MLV polymerase (Promega, USA). Sequence-specific primers were: GAPDH-F: 5′- TGGTGAAGGTCGGTGTGAAC-3′, GAPDH-R: 5′-GCTCCTGGAAGATGGTGATGG-3′; TLR4-F: 5′-ATGGCATGGCTTACACCACC-3′, TLR4-R: 5′-GAGGCCAATTTTGTCTCCACA-3′; PTEN-F: 5′-CCATAACCCACCACAGC-3′, PTEN -R: 5′-AGTCCGTCCCTTTCCAG-3′. Real-time PCR was performed on the IQ5 PCR System (Bio-Rad, USA) with an initial denaturing step at 95°C for 15 seconds, 35 cycles of denaturing at 95°C for 5 seconds, and annealing at 55°C for 30 seconds. Relative expression of real-time PCR products was determined by the ΔΔCt method [13] to normalize target gene expression to that of the housekeeping gene (GAPDH).

### Western blot analysis

Expression of TLR4, PTEN, Akt, and Ser473 phospho-Akt, was detected by Western blotting. Cells were collected and lysed with 1x RIPA lysis Buffer (50 mM Tris-HCl, pH 7.4, 150 mM NaCl, 1% Nonidet P-40, 0.5% deoxycholic acid, 0.1% sodium dodecyl sulfate (SDS), 5 mM EDTA, 2 mM phenylmethylsulfonyl fluoride (PMSF), 20 µg/mL aprotinin, 20 µg/mL leupeptin, 10 µg/mL pepstanin A, 150 mM benzamidine) on ice for 10–15 minutes. Cell debris was pelleted by centrifugation, and protein-containing supernatants were collected. Protein quantification was performed with the BCA method, and SDS-polyacrylamide gel electrophoresis (PAGE) was performed. Proteins were transferred to polyvinylfluoride membranes, probed with the appropriate primary and secondary antibodies, and detected by the ECL+plus™ Western blotting system kit (Amersham, USA). Primary antibodies (1∶1000 dilution) were: mouse anti-TLR4 (ab22048; Abcam, UK), rabbit anti-p-Akt (Phospho-Akt (Ser473), 4060; CST), rabbit anti-Akt (4691; CST), rabbit anti-PTEN (9188; CST), and mouse anti-GAPDH (sc-32233; Santa Cruz Biotechnologies, USA). Secondary antibodies (1∶5000) were: goat anti-mouse IgG (sc-2005; Santa Cruz Biotechnologies) and goat anti-rabbit IgG (sc-2004; Santa Cruz Biotechnologies). Immunoreactivity was visualized by Perfection 3490 photo gel imaging systems (Epson, Japan) and analyzed by Image Pro PLUS. Protein expression was normalized to GAPDH.

### Statistical analysis

All data are represented as mean ± standard deviation (SD). The SPSS statistical software, version 12.0, was used for mean value comparisons of single-factor multiple samples. The homogeneity of variance data were analyzed with the one-factor analysis of variance least squares difference (LSD) test, and the heterogeneity of variance data were analyzed with the Kruskal Wallis rank sum test. Statistical significance was defined as *p*-values less than 0.05.

## Results

### Effect of LPS on lung fibroblast proliferation

**Figure 1:**
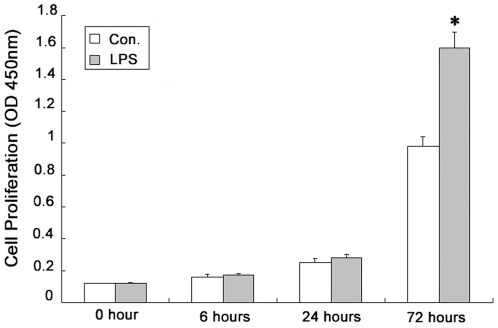
Effect of LPS on lung fibroblast proliferation. DNA synthesis in lung fibroblasts was detected by BrdU assay after LPS challenge at 0, 6, 24, and 72 hours. * *p*<0.05 for percentage of OD_450_ absorbance compared to the control group at the same time point. Columns represent mean values (n = 3) and error bars represent SD.

To investigate the effect of LPS on various stages of lung fibroblast proliferation, we employed the BrdU assay to temporally quantify DNA synthesis in lung fibroblasts in response to LPS challenge. Compared to the control group, the amount of DNA synthesis in lung fibroblasts was increased significantly only at 72 hours after LPS challenge (*p*<0.05) (Fig. 1).

### Expression of TLR4 in lung fibroblasts and its effect on lung fibroblast proliferation

**Figure 2:**
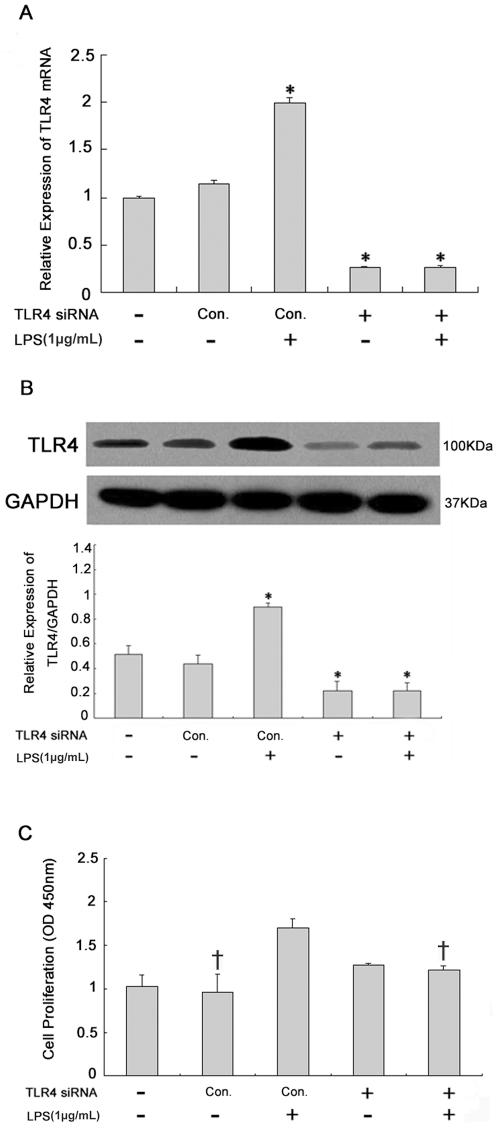
Expression of TLR4 in lung fibroblasts and its effect on lung fibroblast proliferation. TLR4 mRNA (A, real-time PCR) and protein (B, Western blot) expression in lung fibroblasts at 72 hours after 1 µg/mL LPS challenge. Effect of siRNA-mediated knockdown of TLR4 (1×10^8^ TU/mL for 48 hours) on lung fibroblast proliferation (C, BrdU assay). * *p*<0.05 *vs.* negative control group (Column 2); ^†^ *p*<0.05 *vs.* positive control group (Column 3). Columns represent mean values and error bars represent SD. Blots are representative of three independent experiments.

To investigate TLR4 expression in lung fibroblasts and its effect on lung fibroblast proliferation, TLR4 mRNA was knocked down in lung fibroblasts by transfecting TLR4-siRNA-lentivirus. Real-time PCR and Western blot analysis were used to detect TLR4 mRNA and protein expression, respectively, in lung fibroblasts at 72 hours after LPS challenge. The BrdU assay was used to quantify DNA synthesis in TLR4-siRNA-lentivirus-transfected lung fibroblasts. TLR4 mRNA and protein expression in lung fibroblasts increased significantly at 72 hours after LPS challenge (Fig. 2A, B). TLR4 mRNA and protein expression were reduced in lung fibroblasts after TLR4-siRNA-lentivirus transfection, regardless of whether LPS challenge was performed, which indicated a nearly complete inhibitory effect. BrdU assay showed that, compared to unchallenged cells, DNA synthesis increased significantly in lung fibroblasts at 72 hours after LPS challenge (Fig. 2C). However, no differences were observed when the cells were transfected with the TLR4-siRNA-lentivirus.

### Expression of PTEN in lung fibroblasts after LPS challenge or PI3K inhibition

**Figure 3:**
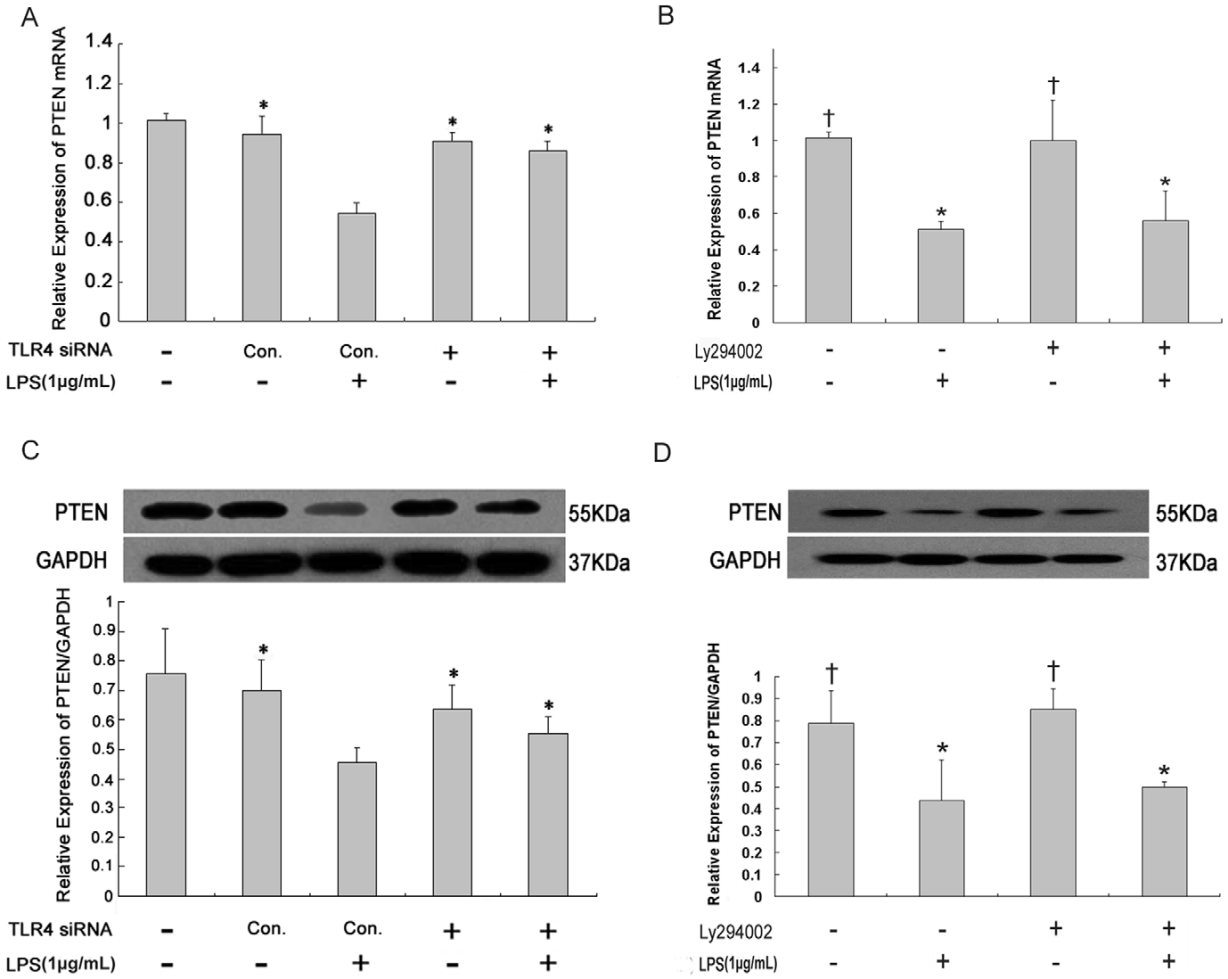
Expression of PTEN in lung fibroblasts after LPS challenge or PI3K inhibition. PTEN mRNA (A and B, real-time PCR) and protein (C and D, Western blot) expression in lung fibroblasts at 72 hours after 1 µg/mL LPS challenge (A, C) or PI3K-Akt pathway inhibition (B, D). siRNA-mediated knockdown of TLR4 (1×10^8^ TU/mL for 48 hours) was used to assess the effect of TLR4 on PTEN expression in lung fibroblasts. Ly294002 (50 µmol/L for one hour) was applied to determine whether PTEN expression is regulated by PI3K activation. * *p*<0.05 *vs.* positive control group (A and C, Column 3). * *p*<0.05 *vs.* negative control group (B and D, Column 1); ^†^ *p*<0.05 *vs.* positive control group (Column 2). Columns represent mean values and error bars represent SD. Blots are representative of three independent experiments.

Real-time PCR and Western blot analysis showed that PTEN mRNA and protein expression was decreased significantly in lung fibroblasts at 72 hours after LPS challenge (Fig. 3A, C). However, no difference was observed after TLR4-siRNA-lentivirus transfection. To elucidate whether PTEN expression is regulated by PI3K activation, LPS-induced inhibition of PTEN expression was investigated in the presence of PI3K inhibitor Ly294002. Ly294002 treatment had no effect on PTEN expression in either normal lung fibroblasts or LPS-challenged lung fibroblasts (Fig. 3B, D).

### Effect of PI3K-Akt pathway and PTEN on lung fibroblast proliferation

**Figure 4:**
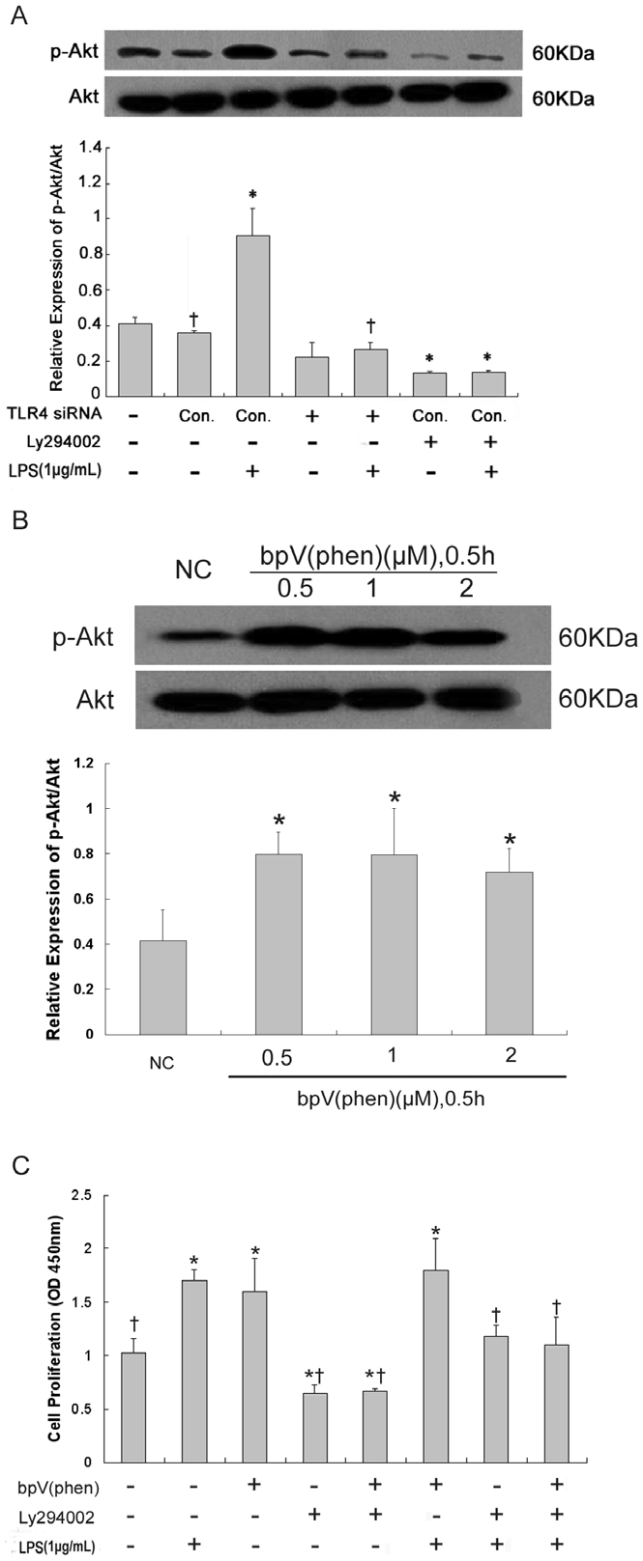
Effect of PI3K-Akt pathway activation and PTEN expression on lung fibroblast proliferation. Activation of PI3K-Akt pathway (A, Western blot) in lung fibroblasts at 72 hours after 1 µg/mL LPS challenge, as detected by expression of Ser473 phospho-Akt. siRNA-mediated TLR4 knockdown (1×10^8^ TU/mL for 48 hours) was used to assess the effect of TLR4 on PI3K-Akt pathway activation. PI3K inhibitor Ly294002 (50 µmol/L for one hour) was used to assess the effect of PI3K-Akt pathway on lung fibroblast proliferation. * *p*<0.05 *vs.* negative control group (Column 2); ^†^ *p*<0.05 *vs.* positive control group (Column 3). Activation of PI3K-Akt pathway in lung fibroblasts (B, Western blot), as detected by expression of Ser473 phospho-Akt after treatment with different concentrations of PTEN inhibitor bpV(phen) for 0.5 hour. * *p*<0.05 *vs.* negative control group (Column 1). DNA synthesis (C, BrdU assay) in LPS-challenged lung fibroblasts after bpV(phen) (1 µM for 0.5 hour) and/or Ly294002 (50 µmol/L for one hour) treatment. * *p*<0.05 *vs.* negative control group (Column 1); ^†^ *p*<0.05 *vs.* positive control group (Column 2). Columns represent mean values and error bars represent SD. Blots are representative of three independent experiments.

PI3K-Akt pathway activation in lung fibroblasts in response to LPS challenge was assessed by Western blot measurement of the Akt phosphorylation product, Ser473 phospho-Akt. PI3K-Akt pathway activation was also used to evaluate the suppression efficiency of PTEN inhibitor bpV(phen) in lung fibroblasts, as described previously [14]. Seventy-two hours after LPS challenge, the BrdU assay was used to quantify DNA synthesis in bpV(phen) and/or Ly294002-treated lung fibroblasts. Compared to unchallenged cells, LPS-challenged lung fibroblasts showed significantly increased phospho-Akt at 72 hours (Fig. 4A). However, TLR4-siRNA-lentivirus transfection had no effect on phospho-Akt expression, either with or without LPS challenge. Phospho-Akt expression was reduced in all Ly294002-treated lung fibroblasts, regardless of whether the cells had been challenged with LPS, which indicated a nearly perfect inhibitory effect. Since the bpV(phen) inhibitory effect on PTEN activation was dose-independent (Fig. 4B), the 1 µM concentration was selected for all subsequent analysis. Both LPS challenge and bpV(phen) treatment led to significantly increased DNA synthesis in lung fibroblasts, as compared to unchallenged cells (Fig. 4C). However, these effects could be reduced or even be reversed by Ly294002 treatment.

## Discussion

Aberrant proliferation of lung fibroblasts has been detected in bleomycin-induced lung fibrosis [15], patients with IPF [11], and LPS-induced ALI and pulmonary fibrosis [4]. Similarly, LPS-challenged C57BL/6 mice presented with cell proliferation in the airways [16]. It is generally accepted that lung fibroblasts develop the property of aberrant proliferation under certain pathological conditions, but the underlying mechanism remains poorly understood. Our present study revealed that LPS can directly induce lung fibroblast proliferation through TLR4 signaling and downstream activation of the PI3K-Akt pathway. Moreover, our results indicated that LPS-activated TLR4 signaling may lead to down-regulation of PTEN, effectively supporting the cell proliferation process.

Recent studies have provided evidence that TLR4 is expressed on lung fibroblasts [6], [17]; however, there is considerable controversy about the effect of LPS challenge on fibroblast proliferation. Yang *et al.* reported that LPS is able to significantly stimulate human skin fibroblast proliferation after more than six days of incubation [18]. Similar results were obtained from cultured human small intestinal lamina propria fibroblasts [19], adventitial fibroblasts [20], [21], 3T6 fibroblasts [22], human periodontal ligament fibroblasts (HPLF) [23], and lamina propria fibroblasts [24]. Human gingival fibroblasts and rat embryo fibroblasts, however, yielded opposite results [25], [26], [27], [28]. Zhang *et al.* demonstrated that LPS can have a dose-dependent inhibitory effect on fibroblast proliferation at 24 hours after LPS challenge [29]. In our present study, LPS challenge of primary cultured mouse lung fibroblasts had no effect on proliferation for up to 24 hours; however, unlike the results from Zhang *et al.*, the lung fibroblasts in our study underwent significant proliferation at 72 hours after LPS challenge. This time point coincides with the period in our previous study when lung fibroblast proliferation and activation were observed in the animal model of LPS-induced ALI and pulmonary fibrosis [4]. Previous clinical research also indicated that lung fibroblast collagen secretion occurred in early stage ALI/ARDS [30]. Therefore, we conclude that LPS can directly promote lung fibroblast proliferation under certain pathophysiologic conditions. It is possible that distinct fibroblast subpopulations [31] or different experimental strategies [32], [33] may account for the inconsistent results reported in the literature. Subsequent studies should be carried out to elucidate the detailed mechanisms through which LPS exerts its effects on the lung fibroblast cell cycle under different physiological and experimental conditions.

TLR4 is an important LPS-specific receptor that is widely distributed among pulmonary cells. LPS-mediated activation of the TLR4 signaling pathway involves several key pathways associated with ALI/ARDS and pulmonary fibrosis, including the PI3K-Akt pathways [34]. Previous studies have shown that activation of TLR4 signaling, including downstream activation of the PI3K-Akt pathway, is strongly correlated to pulmonary cell cycle regulation [35], [36]. Furthermore, the PI3K-Akt pathway has been shown to mediate radiation-stimulated proliferation of normal human lung fibroblasts [7] and hypoxia-induced adventitial fibroblast proliferation [8], and to inhibit endothelin (ET)-1-induced fibroblast apoptosis [37]. The present study revealed that LPS-induced aberrant proliferation of lung fibroblasts is accompanied by overexpression of TLR4 and Akt phosphorylation, indicating activation of the PI3K-Akt pathway. This finding partly agrees with the results reported by Kim *et al.* [7], and those from our previous study [4], [6]. siRNA-mediated TLR4 knockdown and PI3K inhibition by Ly294002 effectively blocked LPS-induced lung fibroblast proliferation, suggesting that LPS can promote lung fibroblast proliferation *via* overexpression of TLR4 and activation of the PI3K-Akt pathway.

In addition, our study also detected down-regulation of PTEN expression in lung fibroblasts after LPS challenge. This effect was inhibited by siRNA-knockdown of TLR4, suggesting that LPS-induced activation of TLR4 signaling could inhibit expression of PTEN in lung fibroblasts. The encoded PTEN protein is a tumor suppressor with phosphatase-like activities that can modulate the PI3K pathway by catalyzing degradation of PI3K-generated PIP3. In this manner, PTEN restrains cell proliferation through inhibiting downstream functions of the PI3K-Akt pathway [38], [39]. PTEN is robustly expressed in normal lung fibroblasts, where it is believed to prevent aberrant proliferation and activation during injury repair [40]; indeed, aberrant fibroblast proliferation and activation in IPF patients was accompanied by low expression of PTEN and activation of the PI3K-Akt pathway [10], [11]. Thus, it is possible that a deficiency in PTEN expression could result in a more durable fibroproliferative response and pathological fibrosis. In our study, we found that the PI3K-Akt pathway is activated and PTEN expression is simultaneously suppressed in response to LPS challenge. In addition, it was determined that the PI3K-Akt pathway activation is downstream of PTEN inhibition, and that the PI3K-Akt pathway represents a key mechanism of the lung fibroblast proliferation process. Therefore, LPS-induced TLR4 signaling may activate the PI3K-Akt pathway by down-regulating PTEN expression in lung fibroblasts. Since the regulatory effect of PTEN on the PI3K-Akt pathway is also closely related to its extent of dephosphorylation and phosphatase activity, further investigation should be carried out to elucidate the role that PTEN plays in LPS-induced lung fibroblast proliferation.

In conclusion, our study provides evidence that LPS can promote lung fibroblast proliferation *via* TLR4 signaling, perhaps accelerating pulmonary fibrosis in the early stages of ALI/ARDS. Furthermore, TLR4-induced down-regulation of PTEN expression and activation of the PI3K-Akt pathway are involved in this process.
